# Targeted Alpha Radiopharmaceutical Therapy and Key Considerations for Nuclear Medicine Technologists

**DOI:** 10.1055/s-0045-1809342

**Published:** 2025-06-03

**Authors:** Julie Bolin, Daryn Groves

**Affiliations:** 1 Department of Nuclear Medicine Technology, GateWay Community College, Phoenix, Arizona, United States; 2 Division of Nuclear Medicine, Department of Radiology, Mayo Clinic, Scottsdale, Arizona, United States

**Keywords:** alpha-particle, beta-particle, molecular target, radiopharmaceutical therapy, targeted α therapy

## Abstract

The use of radionuclides for targeted radiopharmaceutical therapy (RPT) is a rapidly evolving field in nuclear medicine and oncology. With the integration of imaging and therapy, therapeutic nuclear medicine has made remarkable progress in recent years. One particularly promising area of research is the use of α-emitting radionuclides, which possess unique physical properties that provide notable advantages, including the ability to target single tumor cells with high precision. Although the only targeted α therapy (TAT) currently approved by the United States Food and Drug Administration is ^223^ Ra-dichloride for the treatment of castration-resistant prostate cancer with skeletal metastases, a search on clinicaltrials.gov yields a significant number of early- and late-stage clinical trials utilizing 223-Ra, 225-Ac, 211-At, 212-Pb, and 227-Th are in progress, indicating that more TATs are on the horizon. As the prevalence of use for TAT increases, it is important to consider the logistics of TAT administration and the requirements for radiation safety and patient discharge. This review aims to provide a comprehensive overview of the advancements, relevant clinical trials, and logistical considerations associated with targeted α RPT in oncology.

## Introduction

Radiopharmaceutical therapies (RPT) designed for curative treatment, disease control, or palliation are poised to play a significant role in the future of nuclear medicine. RPT involves administering radionuclides that are either linked to tumor-targeting carrier molecules—such as antibodies, peptides, or small molecules—or that naturally accumulate in tumors through cancer cell-specific physiological processes. 1 2 The primary subject of this paper is targeted alpha (α)-therapy (TAT), but there is a discussion of beta (β)-particle RPT highlighting differences in clinical practice. It is important to consider the differences between α- and β-RPT and how these differences impact not only disease management strategies but also the administration requirements, radiation safety protocols, and patient precautions.

## Methods

The authors conducted a comprehensive search of the clinicaltrials.gov database to identify both early- and late-phase clinical trials involving TAT. Among the existing α-emitting radionuclides, only a select few have demonstrated potential for therapeutic applications, particularly in TAT. From this group, the most suitable candidates—namely, 225-actinium ( ^225^ Ac), 211-astatine ( ^211^ At), 212-bismuth ( ^212^ Bi), 213-bismuth ( ^213^ Bi), 212-lead ( ^212^ Pb), 223-radium ( ^223^ Ra), 149-terbium ( ^149^ Tb), and 227-thorium ( ^227^ Th)—were used as key terms in the search for relevant clinical trials. Additional search terms included “targeted alpha therapy,” “alpha,” “RPT,” and “radionuclide therapy.” Trials that had been withdrawn, terminated, suspended, or completed, as well as those with unknown status, were excluded from the results. The search parameters were not further refined by adding other qualifiers such as eligibility criteria, study phase, study type, study results, funding source, or date range. Given the significant number of clinical trials, the authors also performed a literature search to compile radiation safety recommendations relevant to the administration of α-particle RPT. These recommendations are reviewed in the discussion of this article.

## Results

A total of 42 single-center and multicenter clinical trials were identified on clinicaltrials.gov, utilizing the following radionuclides: ^225^ Ac, ^211^ At, ^212^ Bi, ^213^ Bi, ^212^ Pb, ^223^ Ra, and ^227^ Th. Among these, ^225^ Ac and ^223^ Ra were the most frequently employed, featured in 13 and 27 trials, respectively. A summary of ongoing clinical trials involving α-particle therapies, as identified from clinicaltrials.gov, 3 is presented in Table 1 . 3

**Table 1:** Alpha-particle radiopharmaceutical therapy clinical trials

Alpha-particle radiopharmaceutical therapy clinical trials (clinicaltrials.gov—September 13, 2024)							
Isotope	Study title	Cancer type	Target	National clinical trial (NCT)	Study status	Phase	Location
^225^ Ac	Actinium 225 labeled anti-CEA antibody (Ac225-DOTA-M5A) for the treatment of CEA-producing advanced or metastatic cancers	CEA-producing advanced or metastatic cancers	CEA	NCT-05204147	Recruiting	Phase I	1 location, the United States
^225^ Ac	Study of RYZ101 compared with SOC in Pts with inoperable SSTR+ well-differentiated GEP-NET that has progressed following ^177^ Lu-SSA therapy	SSTR+ GEP-NETs	SSTR	NCT-05477576	Recruiting	Phase Ib/3	50 locations in Belgium, Brazil, Canada, France, the Netherlands, Spain, United States
^225^ Ac	Venetoclax and Lintuzumab-Ac225 in AML patients	AML, relapsed or refractory	CD33	NCT-03867682	Recruiting	Phase I/II	5 locations, the United States
^225^ Ac	Study evaluating dosimetry, randomized dose optimization, dose escalation, and efficacy of Ac-225 rosopatamab tetraxetan in participants with PSMA PET-positive castration-resistant prostate cancer	CRPC	PSMA	NCT-06549465	Recruiting	Phase II	1 location, the United States
^225^ Ac	FPI-2265 ( ^225^ Ac-PSMA-I&T) for patients with psma-positive metastatic castration-resistant prostate cancer (mCRPC; AlphaBreak)	mCRPC, previously treated with ^177^ Lu-PSMA RLT	PSMA	NCT-06402331	Recruiting	Phase II/III	6 locations, the United States
^225^ Ac	Targeted alpha therapy with 225 actiniumprostate-specific membrane antigen (PSMA)-I&T of castration-resistant prostate cancer (TATCIST)	mCRPC	PSMA	NCT-05219500	Recruiting	Phase II	2 locations, the United States
^225^ Ac	Prospective registry of targeted radionuclide therapy in patients with mCRPC (reality study)	mCRPC	PSMA	NCT-04833517	Recruiting	Prospective registry	1 location, Germany
^225^ Ac	A study of ( ^225^ Ac)-FPI-2059 in adult participants with solid tumors	NTSR1-expressing solid tumors	NTSR1	NCT-05605522	Recruiting	Phase I	8 locations in Australia and the United States
^225^ Ac	(Ac-225)-PSMA-62 trial in biochemically recurrent and metastatic castration-resistant prostate cancer (mCRPC)	mCRPC, BCR prostate carcinoma	PSMA	NCT-06229366	Recruiting	Phase I/II	1 location, Canada
^225^ Ac	Radioimmunotherapy ( ^111^ Indium/ ^225^ Actinium-DOTA-daratumumab) for the treatment of relapsed/refractory multiple myeloma	Relapsed/refractory multiple myeloma	CD38	NCT-05363111	Recruiting	Phase I	1 location, the United States
^225^ Ac	Study of RYZ101 in combination with SoC in subjects with SSTR+ ES-SCLC	ES-SCLC	SSTR2	NCT-05595460	Recruiting	Phase 1b	12 locations in the United States and Puerto Rico
^225^ Ac	Study of Iomab-B versus conventional care in older subjects with active, relapsed, or refractory acute myeloid leukemia (AML)	AML, relapsed or refractory	CD45	NCT-02665065	Active, not recruiting	Phase III	24 locations in Canada and the United States
^225^ Ac	^225^ Ac-DOTA-Anti-CD38 daratumumab monoclonal antibody with fludarabine, melphalan, and total marrow and lymphoid irradiation as a conditioning treatment for donor stem cell transplant in patients with high-risk acute myeloid leukemia, acute lymphoblastic leukemia, and myelodysplastic syndrome	High-risk AML, ALL, and MDS	CD38	NCT-06287944	Not yet recruiting	Phase I	1 location, the United States
^211^ At	Targeted alpha therapy using astatine (At-211) against differentiated thyroid cancer	Differentiated thyroid cancer (papillary cancer, follicular cancer)		NCT-05275946	Recruiting	Phase I	1 location, Japan
^211^ At	Clinical trial of targeted alpha therapy using (At-211)PSMA-5 for prostate cancer	CRPC	PSMA	NCT-06441994	Recruiting	Phase I	1 location, Japan
^211^ At	^211^ At-OKT10-B10 and fludarabine alone or in combination with cyclophosphamide and low-dose TBI before donor stem cell transplant for the treatment of newly diagnosed, recurrent, or refractory high-risk multiple myeloma	High-risk multiple myeloma-newly diagnosed, recurrent, or refractory	CD38	NCT-04579523	Not yet recruiting	Phase I	1 location, the United States
^203^ Pb/ ^212^ Pb	A first-in-human clinical trial to evaluate an alpha-radiation imaging agent	Neuroendocrine	SSTR	NCT-05111509	Enrolling by invitation	Phase I	1 location, the United States
^212^ Pb	Somatostatin-receptors (SSTR)-Agonist ( ^212^ Pb)VMT-α-NET in metastatic or inoperable SSTR+ gastrointestinal neuroendocrine tumor and pheochromocytoma/paraganglioma previously treated with systemic targeted radioligand therapy	GI NET, PPGL	SSTR	NCT-06427798	Not yet recruiting	Phase I/II	1 location, the United States
^212^ Pb	( ^212^ Pb)VMT-Alpha-NET in metastatic or inoperable somatostatin-receptor positive gastrointestinal neuroendocrine tumors, pheochromocytoma/paragangliomas, small cell lung, renal cell, and head and neck cancers	GI NET, PPGL, SCLC, kidney cancers, or Head and neck cancers (nasopharyngeal carcinoma, olfactory neuroblastoma, sinonasal neuroendocrine carcinoma	SSTR	NCT-06479811	Not yet recruiting	Phase I	1 location, the United States
^212^ Pb	MC1R-targeted alpha-particle therapy trial in adults with advanced melanoma	Melanoma	MC1R	NCT-05655312	Recruiting	Phase I/IIa	9 locations, the United States
^212^ Pb	Targeted alpha-particle therapy for advanced SSTR2 positive neuroendocrine tumors ([212-Pb]-VMT)	Neuroendocrine	SSTR2	NCT-05636618	Recruiting	Phase I/IIa	12 locations, in the United States
^212^ Pb	A safety study of ^212^ Pb-VMT-α-NET in patients with neuroendocrine tumors	Neuroendocrine	SSTR	NCT-06148636	Active, not recruiting	Phase 1	1 location, the United States
^212^ Pb	Targeted alpha-emitter therapy of PRRT naïve and previous PRRT neuroendocrine tumor patients (ALPHAMEDIX02- ^212^ Pb-DOTAMTATE)	Neuroendocrine	SSTR	NCT-05153772	Active, not recruiting	Phase 2	1 location, the United States
^212^ Pb	Safety and tolerability of ^212^ Pb-DOTAM-GRPR1 in adult subjects with recurrent or metastatic GRPR-expressing tumors	mCRPC; HR +/HER2-breast cancer; colorectal cancer; cervical cancer; cutaneous melanoma; NSCLC	GRPR	NCT-05283330	Recruiting	Phase 1	4 locations, the United States
^212^ Pb	A safety study of ^212^ Pb-pentixather radioligand therapy	Carcinoid tumor lung, neuroendocrine tumor of the lung, carcinoma, small-cell lung	CXCR4	NCT-05557708	Not yet recruiting	Phase I	1 location, the United States
^223^ Ra	Phase III radium 223 mCRPC-peace III	mCRPC	Hydroxy-apatite	NCT-02194842	Active, not recruiting	Phase III	64 locations in Belgium, Brazil, Canada, Denmark, France, Ireland, Italy, Norway, Poland, Spain, Switzerland, the United Kingdom
^223^ Ra	Fractionated docetaxel and radium 223 in metastatic castration-resistant prostate cancer	mCRPC	Hydroxy-apatite	NCT-03737370	Active, not recruiting	Phase I	4 locations, the United States
^223^ Ra	Testing the safety of different doses of olaparib given radium-223 for men with advanced prostate cancer with bone metastases	mCRPC	Hydroxy-apatite	NCT-03317392	Active, not recruiting	Phase I/II	22 locations the United States
^223^ Ra	Radium Ra 223 dichloride, hormone therapy, and stereotactic body radiation therapy in treating patients with metastatic prostate cancer	Metastatic prostate carcinoma	Hydroxy-apatite	NCT-03361735	Active, not recruiting	Phase II	1 location, the United States
^223^ Ra	Enzalutamide with or without radium Ra 223 dichloride in patients with metastatic, castration-resistant prostate cancer	mCRPC	Hydroxy-apatite	NCT-03344211	Active, not recruiting	Phase II	3 locations, the United States
^223^ Ra	Impact of DNA repair pathway alterations on sensitivity to radium-223 in bone metastatic castration-resistant prostate cancer	mCRPC	Hydroxy-apatite	NCT-04489719	Recruiting	Observational	4 locations, the United States
^223^ Ra	Testing the addition of radium therapy (radium-223 dichloride) to the usual chemotherapy treatment (paclitaxel) for advanced breast cancer that has spread to the bones	Breast cancer with bone metastases	Hydroxy-apatite	NCT-04090398	Active, not recruiting	Phase II	30 locations in the United States
^223^ Ra	Testing the addition of a new anti-cancer drug, radium-223 dichloride, to the usual treatment (cabozantinib) for advanced renal cell cancer that has spread to the bone, radical study	mRCC with bone metastases	Hydroxy-apatite	NCT-04071223	Recruiting	Phase II	45 locations in the United States
^223^ Ra	Radiation medication (radium-223 dichloride) versus radium-223 dichloride plus radiation enhancing medication (M3814) versus radium-223 dichloride plus M3814 plus avelumab (a type of immunotherapy) for advanced prostate cancer not responsive to hormonal therapy	mCRPC	Hydroxy-apatite	NCT-04071236	Recruiting	Phase I/II	29 locations the United States
^223^ Ra	A study to assess how radium-223 distributes in the body of patients with prostate cancer which spreads to the bones	mCRPC	Hydroxy-apatite	NCT-04521361	Active, not recruiting	Phase 1	12 locations in Austria, France, Israel, Italy, Lithuania, and the United Kingdom
^223^ Ra	Efficacy of Ra-223 in PSMA PET optimally selected patients	mCRPC	Hydroxy-apatite	NCT-05924672	Not yet recruiting	Phase II	1 location, the United States
^223^ Ra	Drug use investigation of Xofigo, castration-resistant prostate cancer with bone metastases	mCRPC	Hydroxy-apatite	NCT-02803437	Active, not recruiting	Postmarketing surveillance (PMS)	Multiple locations, Japan
^223^ Ra	Investigation of radium-223 dichloride (Xofigo), a treatment that gives off radiation that helps kill cancer cells, compared with a treatment that inactivates hormones (new antihormonal therapy, NAH) in patients with prostate cancer that has spread to the bone getting worse on or after earlier NAH	mCRPC	Hydroxy-apatite	NCT-04597125	Active, not recruiting	Phase 4	117 locations in Australia, Austria, Czechia, Finland, France, Germany, Hong Kong, Hungary, Israel, Italy, Republic of Korea, Lithuania, Poland, Russian Federation, Singapore, Spain, Taiwan, Turkey, the United Kingdom
^223^ Ra	Observational study for the evaluation of long-term safety of radium-223 used for the treatment of metastatic castration-resistant prostate cancer	mCRPC	Hydroxy-apatite	NCT-02141438	Active, not recruiting	Observational	68 locations in Argentina, Austria, Belgium, Canada, Columbia, Czechia, Denmark, France, Germany, Greece, Israel, Italy, Luxembourg, the Netherlands, Portugal, Spain, Sweden, the United Kingdom, the United States
^223^ Ra	A study of stereotactic body radiation therapy and radium (Ra-223) dichloride in prostate cancer that has spread to the bones	mCRPC	Hydroxy-apatite	NCT-05133440	Active, not recruiting	Phase II	2 locations, the United States
^223^ Ra	Study evaluating the addition of pembrolizumab to radium-223 in mCRPC	mCRPC	Hydroxy-apatite	NCT-03093428	Active, not recruiting	Phase II	2 locations, the United States
^223^ Ra	68Ga-PSMA PET/CT for Ra223 assessment	mCRPC	Hydroxy-apatite	NCT-04951817	Recruiting	NA	1 location, Taiwan
^223^ Ra	Study of nivolumab in combination with radium-223 in men with metastatic castration-resistant prostate cancer (Rad2Nivo)	mCRPC	Hydroxy-apatite	NCT-04109729	Recruiting	Phase IB	1 location, the United States
^223^ Ra	RAdium-223 and SABR versus SABR for oligometastatic prostate cancers (RAVENS)	mCRPC	Hydroxy-apatite	NCT-04037358	Active, not recruiting	Phase II	1 location, the United States
^223^ Ra	Radium-223 combined with dexamethasone as first-line therapy in patients with M + CRPC (TRANCE)	mCRPC	Hydroxy-apatite	NCT-03432949	Active, not recruiting	Phase IV	1 location, Canada
^223^ Ra	Combination of radium-223 and lutetium-177 PSMA-I&T in men with metastatic castration-resistant prostate cancer (AlphaBet)	mCRPC	Hydroxy-apatite	NCT-05383079	Recruiting	Phase I/II	1 location, Australia
^223^ Ra	Sequencing of Radium-223 and docetaxel in symptomatic bone-only metastatic castration-resistant prostate cancer (RAPSON)	mCRPC; a study to test radium-223 with docetaxel with prostate cancer	Hydroxy-apatite	NCT-03230734	Recruiting	Phase II	15 locations, Italy
^223^ Ra	A study to test radium-223 with docetaxel with prostate cancer	mCRPC	Hydroxy-apatite	NC-T03574571	Recruiting	Phase III	67 locations, Brazil, the Netherlands, Spain, the United States
^223^ Ra	Bipolar androgen therapy (BAT) and radium-223 (RAD) in metastatic castration-resistant prostate cancer (mCRPC; BAT-RAD)	mCRPC	Hydroxy-apatite	NCT-04704505	Recruiting	Phase 2	2 locations, Brazil and the United States
^223^ Ra	SPECT imaging for pharmacokinetics and dosimetry toward treatment optimization (see to treat)	mCRPC	Hydroxy-apatite	NCT-06389097	Not yet recruiting	Observational	No location information
^223^ Ra	Prospective registry of targeted radionuclide therapy in patients With mCRPC (reality study)	mCRPC	Hydroxy-apatite	NCT-04833517	Recruiting	Observational	1 location, Germany
^227^ Th	Study to evaluate the safety, tolerability, pharmacokinetics, and antitumor activity of a thorium-227 labeled antibody-chelator conjugate alone and in combination with darolutamide, in patients with metastatic castration-resistant prostate cancer	mCRPC	PSMA	NCT-03724747	Active, not recruiting	Phase I	5 locations in Finland, the United Kingdom, and the United States.

Abbreviations: ALL, acute lymphoblastic leukemia; AML, acute myeloid leukemia; BCR, biochemically recurrent; CD33, center of differentiation 33; CD38, center of differentiation 38; CD45, center of differentiation 45; CEA, carcinoembryonic antigen; CRCP, castration-resistant prostate cancer; ES-SCLC, extensive stage small cell lung cancer; GEP-NETs, gastroenteropancreatic neuroendocrine tumors; GI-NET, gastrointestinal neuroendocrine tumors; GRPR, gastrin-releasing peptide receptor; HR +/HER2-, hormone receptor-positive, human epidermal growth factor receptor 2 negative; MC1R-melanocortin sub-type 1 receptor; mCRCP, metastatic castration-resistant prostate cancer; MDS, myelodysplastic syndrome; mRCC, metastatic renal cell cancer; NSCLC, non-small-cell lung cancer; NTSR1-neurotensin receptor; PPGL, pheochromocytoma/paraganglioma; PRRT, peptide receptor radionuclide therapy; PSMA. prostate specific membrane antigen; RLT, radioligand therapy; SABR, stereotactic ablative radiation; SCLC, small cell lung cancer; SSAs, somatostatin analogues; SSTR2, somatostatin Receptor subtype 2; SSTR, somatostatin receptor.

While a range of cancer types were evaluated, the three most prominent were metastatic castration-resistant prostate cancer (mCRPC), neuroendocrine tumors (NET), and acute myeloid leukemia (AML). 3 The common therapeutic targets across these trials included hydroxyapatite for mCRPC, somatostatin receptors (SSTR) for NET, and myeloid cell antigens—specifically clusters of differentiation (CD) 33, 38, and 45—for AML or other myelodysplastic diseases. 3

As a result of the established clinical use of ^223^ Ra-dichloride in treating skeletal metastases in mCRPC, several clinical trials with ^223^ Ra are exploring the efficacy of combination therapies with hormone therapy, chemotherapy, immunotherapy, targeted therapy, and stereotactic body radiation. 3 Further, observational and surveillance trials are evaluating the long-term effects of ^223^ Ra-dichloride treatment in mCRPC. 3 For the ^223^ Ra-treatment of skeletal metastases associated with mCRPC, skeletal scintigraphy has been an established pretreatment evaluation; however, clinical trials identified in the search are now investigating the role of PSMA-PET scans for the diagnostic positivity in the progression of ^68^ Ga-PSMA PET versus skeletal scintigraphy. 3 Outside of the evaluation of mCRPC, clinical trials are also investigating ^223^ Ra to treat skeletal metastases in patients with renal cell carcinoma (RCC) and breast cancer. 3 Although ^223^ Ra has long been used for the treatment of mCRPC, its clinical utility has been restricted to skeletal metastases. Clinical trials with ^225^ Ac-PSMA are investigating the treatment of mCRPC with soft tissue or visceral disease progression as well as skeletal lesions.

In the case of AML, the well-characterized nature of cell surface antigens, combined with the radiosensitivity and accessibility of leukemia cells, has driven long-standing interest in radioimmunotherapy (RIT) to complement or replace conventional therapies and improve patient outcomes. While β-particle RIT with radionuclides such as ^131^ I and ^90^ Y has been used to intensify conditioning regimens before allogeneic hematopoietic cell transplantation (HCT), α-particle RIT, using radionuclides like ^225^ Ac or ^211^ At, is being investigated for its precise, potent, and efficient destruction of target cells, with reduced toxicity to surrounding healthy cells. 3 This precision may expand the therapeutic use of α-particle RIT beyond HCT, offering a promising avenue for treatment.

## Discussion

Targeted RPT capitalizes on the distinct differential targeting abilities of molecular vectors to deliver a potent radiation dose directly to cells with higher concentrations of the target. This approach is intended to treat both primary and metastatic cancers in patients identified through molecular imaging, which confirms the presence of biological targets on cancer cells' surfaces or within the vascular or stromal components of metastatic disease. 1 2 The radioisotopes used for RPT emit β- or α-particles, or auger electrons (AE). 2 Table 2 provides a comparison of α and β-particle therapeutic radiation emission characteristics, cytotoxicity, and use. 2 4 5 6

**Table 2:** Comparison of therapeutic radiation emission characteristics, cytotoxicity, and use

	Alpha particles	Beta particles
Particle type	He nucleus (2 protons, 2 neutrons)	High energy electron
Energy	5–9 MeV	0.1–2.2 MeV
Tissue range	50–100 μm	0.05–12 mm
LET	High	Low
Particle pathlength	80 keV/μm	∼0.2 keV/μm
Toxicity	Double Strand DNA breaks Complex multiple clusters Reactive oxygen species	Single-stranded DNA breaks Reactive oxygen species
Bystander effect/crossfire	Yes	Yes
Tumor size	Small neoplasms Micro-metastases	Medium to large tumors
Utilization	Tumors resistant to conventional therapies and β-particle RPT Hypoxic tumors (effective)	Heterogenous tumors Higher volume solid tumor Hypoxic tumors (less effective)
Example nuclides	^223^ Ra, ^225^ Ac, ^212^ Pb, ^211^ At, ^227^ Th	^177^ Lu, ^90^ Y, ^153^ Sm, ^131^ I
Example radiopharmaceuticals	^223^ RaCl _2_ (Xofigo)	^177^ Lu-dotatate (Lutathera) ^177^ Lu-PSMA-617 (Pluvicto) ^90^ Y-microspheres (SIR-Spheres and TheraSpheres) ^153^ Sm Lexidronam (Quadramet) ^131^ I NaI

Historically, targeted RPT primarily relied on β-emitting radionuclides like 131-iodine and 90-yttrium. In recent years, the β-emitting radionuclide 177-lutetium has gained significant clinical relevance, as evidenced by the U.S. Food and Drug Administration (FDA) approvals of ^177^ Lu-PSMA-617 (Novartis, Indiana, approved in 2022) for treating metastatic mCRPC expressing prostate-specific membrane antigen (PSMA), and ^177^ Lu-DOTATATE (Lutathera, Advanced Accelerated Applications, Inc., New Jersey, approved in 2018) for the treatment of somatostatin receptor-positive NET. 1 β-particles are particularly effective in treating medium to large tumors due to their extended particle pathlength and low linear energy transfer (LET). As β-emitters traverse biological tissue, they primarily cause cytotoxicity with single-stranded DNA breaks and the production of reactive oxygen species, exhibiting approximately 500 times lower cytotoxic potency than α-particles. 2 4 While the extended range of β-particles and the resulting cross-fire effect is advantageous for distributing the radiation dose across heterogeneous tumors it can also result in the unintended irradiation of healthy tissue surrounding the tumor site. 2

Recent advancements in targeted radionuclide delivery and the availability of clinically relevant α-emitters have significantly expanded therapeutic possibilities. Although research on α-emitters spans several decades, their current application in targeted RPT represents a pivotal advancement in cancer treatment. Alpha particles are large in size, highly energetic, and have a moderate path length in tissue. 2 Alpha-emitting radionuclides possess the unique ability to selectively destroy targeted tumor cells while sparing adjacent normal tissues due to their high-LET and concentrated radiation deposition, thus making them suitable for small neoplasms, micrometastases, and tumors that are resistant to conventional therapies and β-particle RPT. 2 5 Unlike β-particles, α-particles maintain an almost linear path with destruction occurring all along the path. Efficient cell destruction is accomplished through complex multiple clusters and irreparable double-strand DNA damage. 2 5 Additionally, the interaction of α-particles with water generates reactive oxygen species, which can further react with biomolecules such as proteins, phospholipids, RNA, and DNA, leading to irreversible cellular damage. 5 Due to their particle range, β-emitters are typically believed to produce a stronger cross-fire effect; however, recent studies demonstrating that α-particles can have a significant therapeutic impact on large tumors challenge this assumption. 2 This heightened biological effectiveness, while beneficial for targeting and destroying cancerous cells, also necessitates meticulous handling to prevent unintended exposure. Proper containment, accurate dosimetry, patient monitoring, and adherence to safety protocols are crucial to maximizing therapeutic efficacy and minimizing risks to both patients and healthcare providers.

As TAT clinical trials continue to expand and yield approved therapeutic applications, it is important to consider how this will impact the clinical work for technologists and influence patient interactions and discharge instructions. Alpha particles have a high LET, depositing a significant amount of energy over a short distance, which results in dense ionization and severe biological damage to cells. Due to this characteristic, meticulous handling is essential to prevent unintended exposure. 7 Proper handling of α-emitters is radionuclide-dependent. It is important to consider the decay of progeny for each individual radionuclide when determining shielding requirements, as attenuation of both β and photon radiation may be required with these radionuclides. Using long-handled tools, such as tongs, and shielded syringes or vials helps reduce extremity radiation exposure. 7 Occupational exposure can be further minimized by selecting proper storage methods and shielding. When working with α-emitting radionuclides, depending on the specific radionuclide, lead or plexiglass may be used for shielding. Some α-emitting radiopharmaceuticals, such as ^223^ RaCl _2_ , are supplied as unit dosages and are shipped and stored in specialized containers like the Xofigo Plastic Pig. Other α-emitting radiopharmaceuticals may require dose manipulation and are often shipped and stored in lead containers. 7

Alpha particles, despite their significant ionizing power due to their double-positive charge, have limited penetration because of their large mass. Alpha particles have a very limited range in the air—only a few centimeters—and can be effectively stopped by a thin barrier, such as paper or clothing. Since α-particles require at least 7.5 MeV to penetrate a protective skin layer that is 0.07 mm thick, they do not pose an external radiation risk. 2 However, the primary concern arises from the potential internalization of these particles through inhalation, ingestion, or entry via wounds, which can lead to significant internal damage due to energy deposition in living tissues. To minimize the risk of internal exposure, it is crucial to use appropriate personal protective equipment (PPE), such as gloves (double gloves preferred) and laboratory coats when handling radiopharmaceuticals. 7 Facilities may also require the use of face shields and masks when working with RPT.

Because α-emitting radiopharmaceuticals generate minimal external dose rates, the need for lead shielding or other exposure minimizing shielding in the treatment rooms is minimized. The low external dose rates also facilitate improved patient care, as healthcare staff are not subjected to the same occupational radiation exposure constraints. 7 Administering RPT carries a risk of contamination, particularly since most RPTs are delivered intravenously. To minimize the risk in the event of a spill, absorbent pads should be used to line the injection table and placed around the injection site. 6 Due to the potential hazards associated with α-particles, it is crucial for technologists to implement stringent contamination control measures and decontamination policies, including regular monitoring of workspaces and personal exposure. While a standard Geiger–Muller (GM) detector can be used for monitoring, an α-probe such as ZnS(Ag) scintillator is generally more effective for detecting minimal detectable activity due to its ability to filter out β-particles and photons, thereby achieving lower background levels. 2 7 The counting efficiency of a GM detector depends on various factors, including the type and energy of the radiation being detected. It is essential to review the manufacturer's specifications to ensure accurate energy sensitivity.

A few key administration considerations are crucial, particularly due to the increased risks associated with needle sticks and skin contamination during IV administrations. These include always ensuring IV patency, utilizing ultrasound guidance for insertion when available, and regularly checking patency to prevent IV infiltrations. The use of needleless connectors is encouraged when possible, along with the placement of absorbent pads or gauze at connection sites to prevent leaks or spills on the patient's skin during connection.

The Nuclear Regulatory Commission (NRC) requirements related to needlestick injuries and skin contamination are specific to the United States. It is important to note that regulatory frameworks vary by country, and each nation may have its own standards and reporting protocols for managing radiation-related occupational exposures. Accidental needle sticks and skin contamination during treatments are classified as special events by the NRC and require an immediate and appropriate response due to the potential risk of radioactive material intake. 7 Prompt decontamination of the affected area, along with continuous monitoring, is critical. 7 NRC Regulatory Guide 8.9 outlines detailed procedures for assessing radioactive material intake through bioassay and determining if further investigation is necessary. 7 In situations requiring specialized monitoring, any suspected intake of radioactive material should be thoroughly evaluated to ensure the proper response is taken to mitigate potential risks.

A study published by Serencsits et al evaluated external exposure rates in patients treated with ^223^ Ra, ^225^ Ac, and ^227^ Th. 7 As expected, the external exposure rates for patients treated with α-emitting radionuclides were found to be minimal, with median dose rates of less than 0.5 μSv/hour measured at a 1-m distance. Considering the low external dose rates, it is highly unlikely that any patient would expose a member of the public to more than 1 mSv of radiation—the threshold outlined in the U.S. NRC regulatory guide 8.39 for the release of patients treated with radioactive materials. 7 This aspect of α-emitting radionuclide therapy offers a distinct advantage, eliminating many of the restrictions commonly associated with other forms of radiopharmaceutical treatment.

In most jurisdictions, patients can resume their normal activities immediately after treatment without the need for radiation safety precautions; however, caution is still advised when handling specimens containing bodily fluids to prevent accidental ingestion of radioactive material. 7 To minimize any potential risk to the public or household members, specific guidelines regarding the management of bodily fluids are provided to patients upon discharge (see Table 3 ). These recommendations include sitting while using the restroom, thoroughly washing hands after any contact with bodily fluids, and promptly cleaning up any spills of vomit or bodily fluids. These precautions are advised for 1 week following therapy, although most radioactive excretion occurs within the first 72 hours. Beyond this period, the remaining levels of radioactivity are negligible and pose no significant risk of exposure to others. 7

**Table 3:** Radiation safety—sample patient discharge instructions

Radiation safety precautions following your radiopharmaceutical therapy
There are no restrictions regarding personal contact. It is safe for you to interact with and be in close proximity to people The therapeutic medication may be eliminated in bodily fluids such as blood, feces, vomit, urine, saliva, or semen. Because of this, it is important to use the following precautions for 1 wk following administration • Stay well hydrated • Practice good personal hygiene, such as frequent handwashing, especially after using the restroom • Sit when using the toilet to minimize urine contamination. Flush the toilet two times after each use • Wear disposable gloves when handling any bodily fluid or when handling items contaminated with bodily fluid. This includes handling towels, clothes, and linens. Wash these items separately from the regular laundry • Clean up any spills of bodily fluids promptly with disposable materials and wash hands thoroughly with soap and water If you need to seek medical care or need to provide a urine or blood sample • Inform your healthcare provider of your treatment with α-particle radiopharmaceutical therapy Contraception • Women of childbearing potential must use a barrier contraception and a second form of highly effective contraception while receiving the study drug and for 6 mo following their last dose • Sexually active male subjects must use a condom during intercourse while receiving the study drug and for 3 months after the last dose of the study drug and should not father a child during this period

Taking into account the distinct differences between α- and β-RPTs, medical institutions must develop specific competencies and clear guidelines as part of a comprehensive RPT management program. Institutions offering both α- and β-RPT will require well-designed and streamlined standard operating procedures (SOPs) to address the unique demands of these therapies. Key considerations for such a program include scheduling, staff utilization, room requirements, and radiation safety practices, all of which are significantly impacted by the characteristics of α- and β-particle therapies. Table 4 outlines practical considerations for implementing a radiopharmaceutical management program, while Table 5 provides a comparative analysis of practical considerations specific to α- and β-RPTs. 7 These tools aim to guide institutions in creating efficient, safe, and effective RPT programs that meet the operational and regulatory requirements for both therapy types.

**Table 4:** Practical considerations for administering radiopharmaceutical therapy

Category	Recommendation
Regulatory compliance	• Ensure all processes adhere to local, state, and federal regulations • Maintain licenses and certifications for personnel and facilities • Ensure compliance with international guidelines, if applicable
Staff training and competency	• Provide initial and ongoing training in radiation safety and radiopharmaceutical therapy protocols • Certify staff competency through periodic evaluations and refresher courses • Encourage participation in continuing education on advancements in radiopharmaceutical therapy
Standard operating procedures (SOPs)	• Develop and document detailed SOPs for all aspects of therapy, including preparation, method of administration (infusion, gravity method, combined), and disposal • Regularly review and update SOPs based on new guidelines or technologies
Patient safety	• Verify patient identity and therapy prescriptions before each procedure • Use bar code medication administration (BCMA) technology to scan both the patient and the medication prior to administration. This ensures accurate verification and significantly reduces the potential for incorrect administration. Comparable systems—such as electronic medication management (eMM) and closed-loop medication administration—are similarly employed to enhance safety and accuracy in radiopharmaceutical delivery • Use two-person identification of the dose • Ensure venous access • Use a skin barrier to prevent accidental skin contamination during administration
Staffing needs	• Evaluate staffing requirements to ensure adequate coverage during therapy administration, including preparation and monitoring. Additionally, account for the time required for controlled area preparation and room turnover to ensure compliance with safety and regulatory standards • Consider utilizing two nuclear medicine technologists (NMT) for handling and documentation: one NMT is deemed “hot” and wears full personal protective equipment (PPE), the second NMT double checks everything and is the scribe for any labels/computer entry, etc
Radiopharmaceutical handling	• Use appropriate shielding devices (syringe shields) and containment when handling radiopharmaceuticals • Shielding requirements should be individualized to the radioisotope and take into account the progeny decays • Wear personal protective equipment, such as gloves (double gloves), goggles or face shields, laboratory coats, and dosimeters
Radiation safety	• Monitor radiation exposure using dosimeters • Implement competency determination for donning and doffing PPE • Implement a color-coded system for isotopes to clearly differentiate between radiopharmaceuticals and minimize the risk of administration errors • Survey NMTs for contamination prior to entering the therapy room. This ensures that any detected contamination originates from the therapy procedure rather than preexisting sources, such as Tc-99, thereby maintaining a controlled and traceable environment
Recordkeeping	• Maintain detailed and accurate records of all therapies, training, and audits
Patient follow-up	• Monitor therapeutic outcomes and side effects through systematic follow-ups
Audits and reviews	• Conduct routine quality assurance audits to identify areas for improvement
Emergency preparedness	• Establish and communicate protocols for handling spills or accidental exposures • Train staff in spill management and emergency procedures • Have spill kits available
Continuous improvement	• Regularly evaluate data to refine protocols and improve program outcomes

**Table 5:** Practical considerations for staff performing alpha versus beta radiopharmaceutical therapy

Aspect	Alpha radiopharmaceutical therapy	Beta radiopharmaceutical therapy
Radiation shielding	Should be individualized to the radionuclide and account for progeny decay types	Requires shielding for β-particles and potentially gamma photons. Bremsstrahlung radiation should be considered in determining shielding requirements
Personal protective equipment (PPE)	Double gloves, laboratory coats, face shields, and masks to avoid contamination	Double gloves, laboratory coats, face shields, and masks to avoid contamination. May utilize lead aprons
Handling and containment	Use tongs, shielded syringes, and specialized containers (e.g., Xofigo Pig)	Use tongs, shielded syringes, and containment boxes. Use low atomic number attenuation materials with pure β-emitters
Spill management	Immediate containment; use absorbent materials and α detectors preferred (e.g., ZnS scintillators if available and Geiger–Muller survey meters)	Contain spills quickly and monitor using β-detectors and Geiger–Muller survey meters
Monitoring	Use α-probe detectors to monitor contamination	Use Geiger–Muller detectors or β-probes for radiation monitoring
Exposure risk	Primary concern is internal contamination (inhalation, ingestion, and wounds)	Risk of both internal and external exposure, especially to extremities
Patient interaction	Minimal external dose rates; close patient contact is safe during and after therapy	External dose rates require distance precautions during procedures. May require hospitalization of patients in units specifically designed and equipped with special shielding and contamination control
Patient discharge	Basic hygiene precautions (e.g., double flushing toilets and proper handwashing)	May involve stricter guidelines to minimize external exposure to others. Extent of isolation and precautionary instructions are dependent on the radioisotope and quantity administered
Special considerations	Assess decay progeny for shielding requirements (α, β, and photon emissions)	Consider β-particle range and potential radiation exposure during administration

## Conclusion

Targeted α-therapy (TAT) is emerging as one of the most promising innovations in cancer treatment. By delivering highly cytotoxic doses directly to cancer cells while sparing surrounding healthy tissue, TAT offers a distinct advantage over other forms of radiation therapy. Utilizing the high LET and short path length of α-particles, this approach enhances therapeutic efficacy and reduces potential side effects. With appropriate PPE, training, and radiation detection protocols in place, α-emitting radionuclides can be safely handled and administered in clinical settings, minimizing contamination risks and ensuring the safety of healthcare professionals and the public. Additionally, patient release guidelines for TAT often require only basic hygiene precautions to prevent accidental ingestion or inhalation of radioactive material by others, allowing patients to resume normal activities without the strict radiation restrictions seen in other therapies. The field of TAT is widely regarded as one of the most exciting areas of innovation in cancer therapy today. Early- and late-stage clinical trials for NET and metastatic prostate cancer are already progressing and industry support is advancing new TAT concepts. As research and development continue, TAT, along with β-particle RPT, is expected to significantly broaden the range of options available for cancer treatment, including effective combination therapies to maximize outcomes.
